# Tracking and modeling the movement of Queensland fruit flies, *Bactrocera tryoni*, using harmonic radar in papaya fields

**DOI:** 10.1038/s41598-024-67372-4

**Published:** 2024-07-30

**Authors:** Anika L. Hurst, Allison L. O′Brien, Nicole D. Miller, Allysen M. Welty Peachey, James M. Yoder, Stefano G. De Faveri, Jodie Cheesman, Nicholas C. Manoukis, Matthew S. Siderhurst

**Affiliations:** 1 Department of Biology, Eastern Mennonite University, 1200 Park Road, Harrisonburg, VA 22802 USA; 2 Department of Biology and Environmental Science, Bridgewater College, 402 East College Street, Bridgewater, VA 22812 USA; 3 Department of Chemistry, Eastern Mennonite University, 1200 Park Road, Harrisonburg, VA 22802 USA; 4 Department of Agriculture and Fisheries, Queensland Government, 28 Peters Street, Mareeba, QLD 4880 Australia; 5 Daniel K. Inouye US Pacific Basin Agricultural Research Center, United States Department of Agriculture, Agricultural Research Service, Hilo, HI 96720 USA; 6 Department of Agriculture and Fisheries, Queensland Government, 26-40 Delancey Street, Cleveland, Queensland 4163 Australia

**Keywords:** Directional movement, Modeling, Field tracking, Movement simulation, Hidden Markov models, Fractal analysis, Agroecology, Entomology

## Abstract

Determining movement parameters for pest insects such as tephritid fruit flies is critical to developing models which can be used to increase the effectiveness of surveillance and control strategies. In this study, harmonic radar was used to track wild-caught male Queensland fruit flies (Qflies), *Bactrocera tryoni,* in papaya fields. Experiment 1 continuously tracked single flies which were prodded to induce movement. Qfly movements from this experiment showed greater mean squared displacement than predicted by both a simple random walk (RW) or a correlated random walk (CRW) model, suggesting that movement parameters derived from the entire data set do not adequately describe the movement of individual Qfly at all spatial scales or for all behavioral states. This conclusion is supported by both fractal and hidden Markov model (HMM) analysis. Lower fractal dimensions (straighter movement paths) were observed at larger spatial scales (> 2.5 m) suggesting that Qflies have qualitatively distinct movement at different scales. Further, a two-state HMM fit the observed movement data better than the CRW or RW models. Experiment 2 identified individual landing locations, twice a day, for groups of released Qflies, demonstrating that flies could be tracked over longer periods of time.

## Introduction

Characterizing the movement parameters of pest insects is critical to developing models which can be used to increase the effectiveness of surveillance and control strategies. Tracking individual insect movements in the field, when possible, allows the determination of movement parameters such as step-distance and turning angles in natural environments^1^. For the Queensland fruit fly (Qfly), *Bactrocera tryoni* (Froggatt) and tephritid fruit fly movement more generally, mark-release-recapture^2^, flight mills^3^, and visual observations have been used to study fly movements, however, none of these techniques give a full picture of movement in nature.

Tracking devices previously employed to study individual insect movement including radio frequency identification (RFID), radio telemetry (RT), and harmonic radar (HR)^4^. Relatively few dipteran spp. have been studied using tracking devices^4^ as flies are generally small- to medium-sized insects requiring small, light tags. In contrast, Hymenoptera, including honey bees, bumble bees, and wasps, have been relatively frequent subjects of individual tracking likely due to the ability of these insects to fly with an attached tag^4^.

To our knowledge, all previous dipteran tracking studies have utilized HR (Tachinidae^5^, Sarcophagidae^5^, Tephritidae^1,6–9^, Glossinidae^10^), with studies on tephritids including work on *Bactrocera minax*^6–9^, and *Zeugodacus cucurbitae*^1^. HR tags have the advantage of generally being much lighter than RT tags although they generally have a shorter detection range^4,11^. There are two components of an HR system, 1) a radar transceiver unit, which both emits a directional microwave signal and ‘listens’ for a reflected signal at twice the broadcast frequency, and 2) a diode tag that receives the original microwave signal and reemits a frequency-doubled signal^12^. HR units can be stationary ground-based^11^ or mobile, which includes handheld units^5,13^. Previous studies have tracked insects, including several fly species^5^, using handheld HR units manufactured for avalanche rescue by the RECCO corporation^12,14,15^.

Qfly is a major pest of horticultural crops in eastern Australia, attacking a wide range of fruit crops^16–18^ and restricting interstate and international trade^19,20^ To counter the threat of invasive tephritid fruit flies, such as Qfly, government agencies deploy trapping networks for early detection of tephritid pests^21^. When tephritids are detected, delimitation and quarantine efforts are triggered as a regulatory response and to avoid establishment^22–24^. These involve applying measures such as increased trapping, insecticide application, protein baiting, male annihilation, and sterile insect technique over an affected area, but the size of this area is often difficult to set^25,26^. This difficulty arises for several reasons including that the location of the introduction is unknown, as well as because the spread of a population depends on multiple factors such as the length of time since the incursion and, critically, the dispersal ability of the pest fly.

At the farm level, until relatively recently, control of *B. tryoni* was primarily accomplished via spraying the insecticides dimethoate and fenthion. However, with restrictions on these chemicals, integrated pest management (IPM) has become more important for Qfly control^18,27^. IPM employs a combination of control methods, including cultural, biological, and chemical approaches to manage pest Qfly populations while minimizing negative environmental impacts. For tephritids, IPM control strategies may include field sanitation, the use of semiochemicals for both monitoring and control, male annihilation technique (MAT), sterile insect technique (SIT), protein baits laced with toxicants, biological control via natural enemies such as parasitoids, and areawide management (applying IPM techniques over a large geographical area)^28,29^.

While there is some information about Qfly population dispersion in nature, much less is known about the movements of individual Qflies in their environment^25,30^. Determining flight movement parameters for individual *B. tryoni* can improve large-scale surveillance and invasion counter-measures as well as farm-level IPM strategies. Movement data may also provide insights into how to optimize IPM control strategies as has been done for the brown marmarotid stink bug, *Halyomorpha halys*^31^ and the spotted wing drosophila, *Drosophila suzukii*^32^. Additionally, fly movement data will allow better modeling of pest populations to understand potential pest distribution, quarantine deployment, optimizing trapping networks, and predicting pest outbreaks^33^. These goals might be attained by optimizing detection and control measures through analysis of individual-based (“agent-based”) models. Agent-based models are computational simulations of unique and autonomous individuals (agents) that interact with the local environment and other agents^34^.

Fruit flies may move to find food, mates, oviposition sites, and protection in dense vegetation. This movement may be over short distances, such as within crop fields and orchards, or long range via ship ports and human-assisted movement in urban areas. Individual Qfly movement data enables detailed modeling of populations of this species, which will enhance our understanding of potential pest distribution, aid quarantine deployment, optimize trapping networks/toxicant baits placement, predict pest outbreaks, and potentially improve SIT^26,35–38^. Previous agent-based simulations have addressed management and eradication of tephritids^35,37,38^ including Qfly^39,40^. Models focused on trapping particularly benefit from realistic movement modeling^36,41,42^, since target insect movement contributes substantially to capture probability^26,43^. Spatially explicit models of fruit fly movement would benefit especially from real-world estimates of certain parameters: Fly step-distances, flight directionality, and movement rates.

Diffusion models have long been used to quantify insect movement^44^. They are mathematically tractable and yield modeled distributions of individuals, but they don’t explicitly model the movement process and so may not adequately reflect distributional outcomes—especially the effect of rare, longer range or ballistic movement^43,45,46^. With the ability to track the movement of individual flies, it is possible to test the distributional outcomes of diffusion models, particularly the common assumption that flies move in random directions. Qfly have been reported to disperse in random directions^47^ while there has also been the suggestion that they are likely to exhibit some degree of correlated directional flight on the level of individual fly movements^48^. Both simple random walk (RW, i.e., Brownian) movement^49,50^, that is turning angles with a random distribution, and correlated random walk (CRW) movement^1,51^, in which the turning angles of successive steps show some degree of correlation, have been observed in dipterans and tephritids more specifically.

This study aimed to determine movement parameters for wild male Qflies in a papaya field in northern Queensland, Australia using HR tracking. To do this, two field experiments were conducted. The first involved nearly constant fly observation with flies disturbed to induce movement. Data from this experiment was used to model Qfly movement. The second used intermittent determination of Qfly positions over the course of several days to gather information on the distributional outcome of individual movement events.

## Results

**Figure 1:**
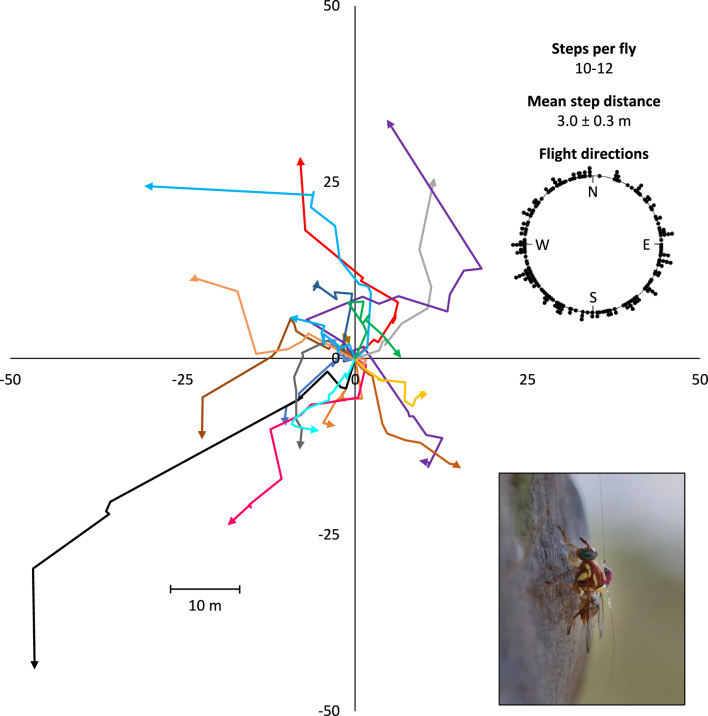
HR tagged *Bactrocera tryoni* flight tracks for Experiment 1 (induced movement). Colored arrows represent a series of 10–12 flights for a single tagged fly. When all flights were taken together (inset top right), flight directions were homogeneous showing no directionality (*P* = 0.078, Rayleigh test; *P* = 0.097, Hermans-Rasson test).

Experiment 1 tracked male Qflies using nearly constant fly observation. Key data collected during Experiment 1 include flight directions, step-distances, and turning angles. Analysis of all flights from Experiment 1 together (all flights combined) showed that flight directions were homogeneous showing no directionality (*P* = 0.078, Rayleigh test; *P* = 0.097, Hermans-Rasson test) (Fig. 1). However, a Watson-Williams test showed that flight angle means for each fly were not homogenous (*F* = 17.681, df1 = 19, df2 = 187, *P* < 0.001) showing that mean flight directions varied between flies. Additionally, non-random flight directionality was detected in 7 of the 20 flies using either Rayleigh test (*P*-values ranged from 0.780 to < 0.001) or Hermans-Rasson test (*P*-values ranged from 0.764 to 0.001) (Fig. S1).

**Figure 2:**
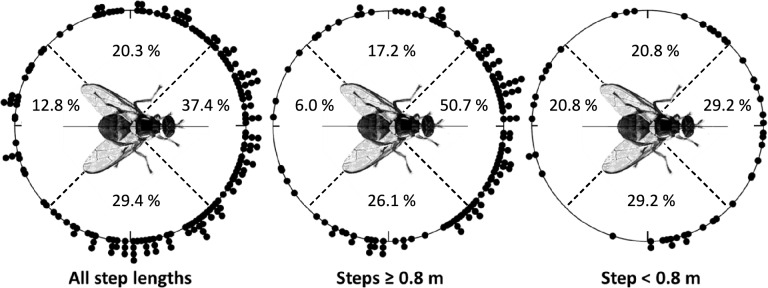
Combined turning angles of HR tagged *Bactrocera tryoni* for Experiment 1. Combined turning angles for all step-distances (concentration of 0.61) (**A**) and steps greater than or equal to 0.8 m (**B**) were non-random by both Rayleigh and Hermans-Rasson tests, showed no right-left bias, but indicate a pronounced bias towards moving within 90° left or right of the directly previous flight. Conversely, combined turning angles for steps under 0.8 m (**C**) were random by both Rayleigh and Hermans-Rasson tests, showing no directional movement bias.

Combined turning angles for all step lengths (Fig. 2A) and steps greater than or equal to 0.8 m (Fig. 2B) were non-random by both Rayleigh and Hermans-Rasson tests (*P* < 0.001 for all tests), showed no right-left bias for either set on turning angles (*P* > 0.05, chi-squared tests), but indicate a pronounced bias towards moving within 90° left or right of the directly previous flight (*P* < 0.001, chi-squared tests). Conversely, combined turning angles for steps under 0.8 m (Fig. 2C) were random by Rayleigh (*P* = 0.075), Hermans-Rasson (*P* = 0.134), and chi-squared (*P* = 0.721) tests, showing no directional movement bias.

**Figure 3:**
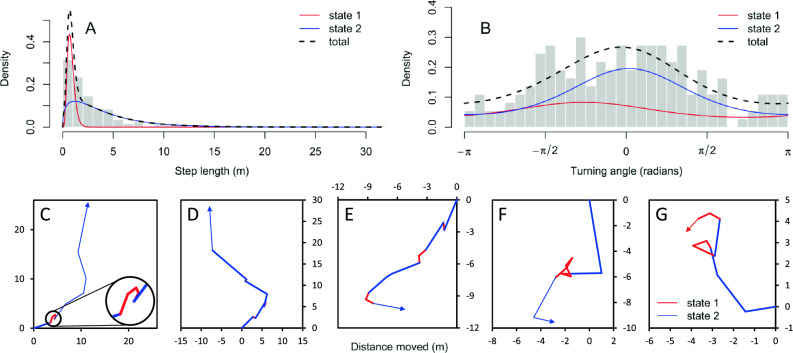
Hidden Markov model of *Bactrocera tryoni* movements in Experiment 1. The distributions of step lengths (**A**) and turning angles (**B**) are shown for the two-state model. State 1 (shown in red) is comprised of fly movements with shorter step lengths (**A**) and more random turning angles (**B**) while state 2 movements are generally longer (**A**) and more show a greater propensity to maintain a directional heading over multiple steps (**B**). Grey bars show the proportion of fly movements for a given step length (**A**) or turning angle (**B**). Example flight tracks are shown in C and D with state switching illustrated.

The observation that shorter step lengths appear to correlate with more random turning angles prompted an analysis with a two-state HMM (Fig. 3). State 1 showed a step-distance of 0.8 ± 0.3 m (mean ± standard deviation) with a mean turning angle of -0.82 radians and a concentration of 0.46 while state 2 had a mean step-distance of 4 ± 3 m with a mean turning angle of 0.05 radians and a concentration of 0.77 (Fig. 3A,B). The maximum log-likelihood for the 2-state HMM was -732.1. Observations indicate that state 1 steps generally represent within-tree movement while state 2 steps were generally between papaya trees.

**Figure 4:**
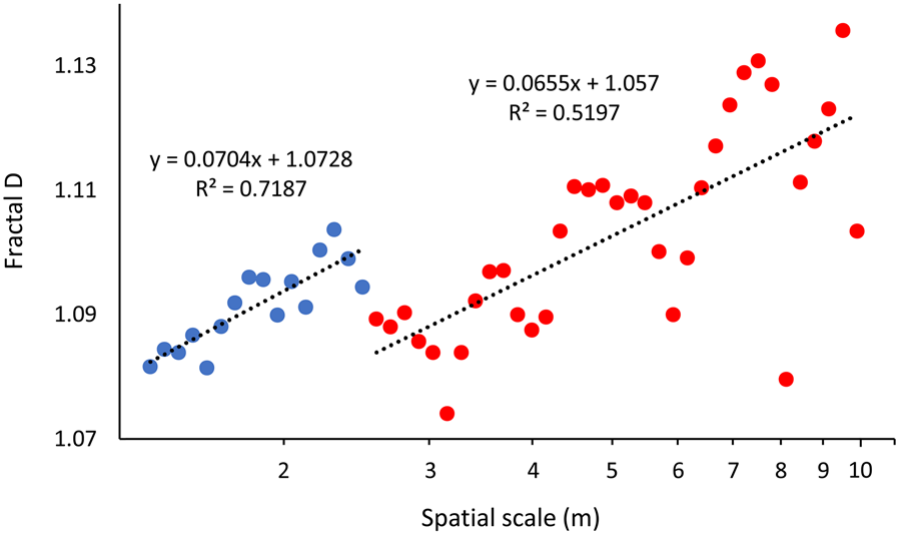
Combined fractal dimensions from eighteen of the observed *Bactrocera tryoni* movement paths (two shorter paths were excluded). Fitting a discontinuous two-phase model showed a change point in the fractal dimension curve at a spatial scale of 2.48 m (black dotted lines show the two linear estimations). This suggests that Qflies in papaya fields move qualitatively differently at spatial scales < 2.48 (blue dots) and > 2.48 m (red dots). Linear regressions were performed on log transformed data.

An analysis of fractal dimension vs. spatial scale using a discontinuous two-phase linear model showed a change point in the fractal dimension at a scale of 2.48 m (maximum of likelihood ratio statistic = 12.03, *P* = 0.039, Fig. 4). The R^2^ value for the single linear regression (R^2^ = 0.49) is lower than the two values from the discontinuous two-phase model (R^2^ = 0.72 and 0.52). Additionally, fitting a continuous two-phase model (assuming both slopes ≠ 0) did not find a significant change point (maximal statistic = 6.35, threshold = 3.15 m, *P* = 0.058).

**Figure 5:**
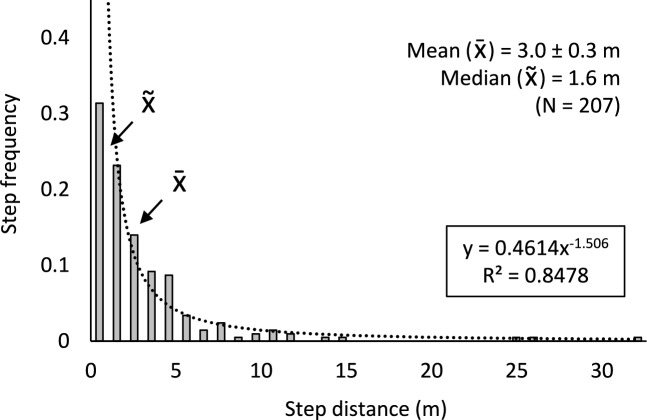
*Bactrocera tyroni* flight step-distances for Experiment 1. Step-distances were categorized into 1 m intervals for this analysis.

**Figure 6:**
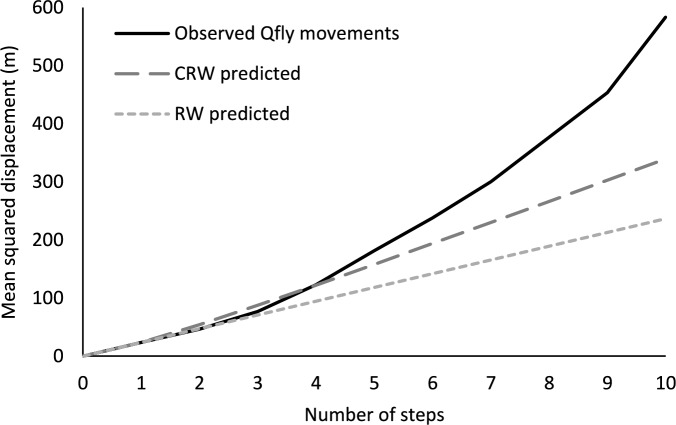
Mean squared displacement (m) distances by number of consecutive movement steps for *Bactrocera tyroni* in Experiment 1.

Overall, the mean step-distance for Experiment 1 was 3.0 ± 0.3 m with a median of 1.6 m (N = 207, Fig. 5). Movement paths (10–12 steps) ranged in length from 7.7 to 75.7 m (31 ± 4 m, mean ± SE). Step-distances for Experiment 1 are well described by the power equation, step freq. = 0.4614 × step dist.^−1.506^ (*R*^*2*^ = 0.8478) (Fig. 5). Step-distances were categorized into 1 m intervals for this analysis. Analysis of mean squared displacement (Fig. 6) show that Qfly movements conform to the expectations of a RW or CRW walk model up to 4 steps. For steps 5 and greater, the mean squared displacement is greater than what would be predicted by either model.

**Figure 7:**
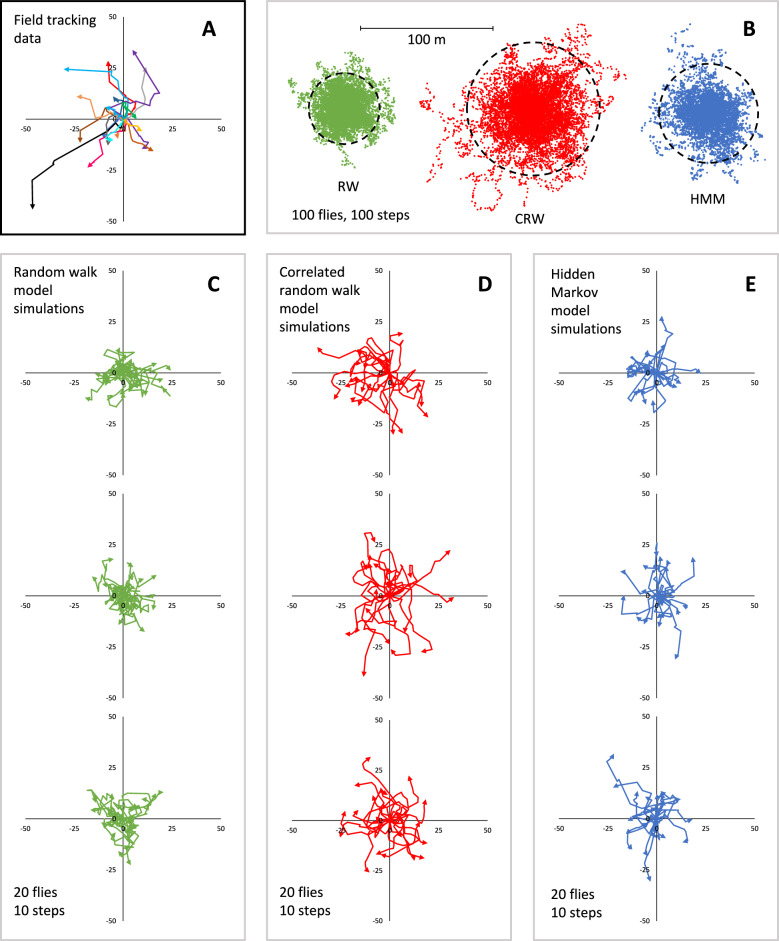
Comparisons between *Bactrocera tyroni* field tracking data (**A**) and model simulations. Example simulations of 100 flies each taking 100 steps based on three movement models (**B**). Dotted black lines show circles containing 95% of all steps. Example simulations of 20 flies each taking 10 steps, roughly matching the field tracking data are shown for (simple) random walk (Brownian) (**C**), correlated random walk (**D**), and hidden Markov (**E**) models.

**Figure 8:**
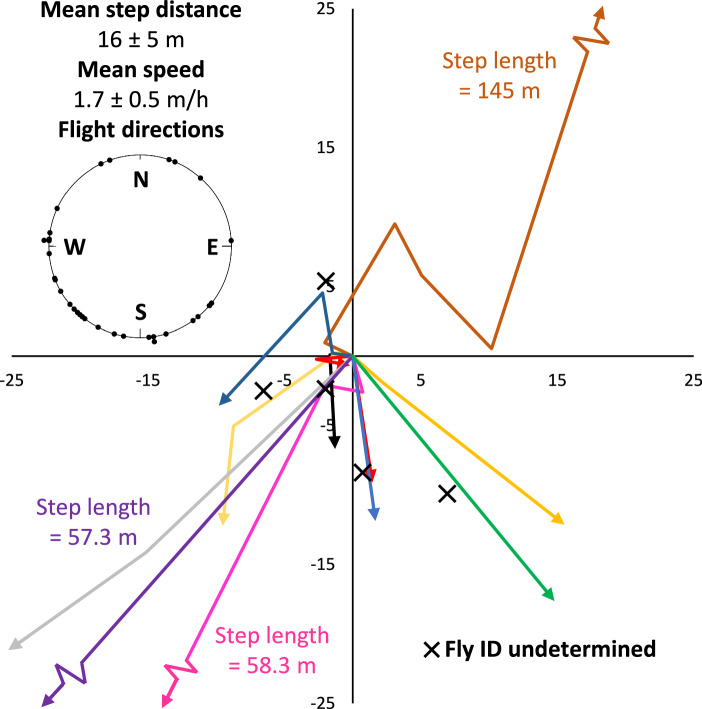
HR tagged *Bactrocera tryoni* flight tracks for Experiment 2 (natural movement). Colored arrows represent a series of movements for a single tagged fly. Black Xs show locations where the detected fly could not be visually identified. When all flights were taken together (inset bottom right), flight directions were not homogeneous showing directionality (*P* = 0.004, Rayleigh test; *P* = 0.018, Hermans-Rasson test).

Visual inspection of field tracking data (Fig. 7A) compared to example model simulations (Fig. 7C–E) suggest that a RW model provides the worst model fit based on the dispersal patterns alone. This observation is supported by Akaike information criterion (AIC) values evaluating the model fit for the movement data of each individual Qfly tracked in Experiment 1 (Table S1). For 19 out of the 20 Qflies tracked, the AIC value was higher with a RW than with a CRW indicating better model fits with CRW. For fly T3 modeled by CRW, the AIC value returned was infinite. When all Qfly movements are combined in a model, the AIC for HMM (1486.18) is slightly lower than for CRW (1518.58) suggesting HMM provides a marginally better model fit. Simulations of 100 flies taking 100 steps each (Fig. 8B) showed the maximum distance moved from the origin were longest with CRW, followed by HMM, with the shortest maximum distances predicted by RW modeling (Table S1). This trend holds for the mean radius about the origin encompassing both 95% and 50% of movements (Table S2).

Experiment 2 used intermittent determination of Qfly positions over the course of several days to gather information on the distributional outcome of individual movement events. Analysis of all flights from Experiment 2 together (all flights combined) showed that flight directions were not homogeneous (*P* = 0.004, Rayleigh test; *P* = 0.016, Hermans-Rasson test) showing flight bias toward the southwest (Fig. 8). Turning angles and comparisons between the mean flight directions of individual flies were not calculated for Experiment 2 due to the small number of observations. The mean total distance moved per fly for Experiment 2 was 16 ± 5 m. The mean speed of movement recorded was 1.7 ± 0.5 m/h. Flies were successfully located up to almost 26 h after initial release (Fig. 8). No further data were collected due to field access issues. Experiment 2 was intended to run over a longer period of time but was cut short on both attempts when the papaya fields were unexpectedly sprayed with fungicides.

Qfly males that were immediately frozen after capture had a mean mass of 12 mg. Qfly males frozen after being caged for 24 h (or after recapture from the field) were found to have a mean mass of 11 mg. Tag to fly mass ratios (percentages) are therefore 6.7% and 7.3%, respectively.

## Discussion

Detection and control efforts against pest tephritids such as Qfly often rely on understanding how these species move within the landscape. Most studies that address this question have used MRR and report the distances flies move between the release point and traps set at regular distance intervals. On the longer range of distances, MacFarlane et al.^52^ reported a single Qfly that moved 94 km while Fletcher^47^ claimed movement up to 22.7 km. However, the vast majority of Qfly dispersal distances do not exceed 600 m^25,30,53^. Similar movement distances have been reported for other pest tephritids such as *B. dorsalis*^54–57^, *B. tryoni*^2,47,52,58^, *B. latifrons* (Hendel)^59^, *B. oleae*^60^, *Z. cucurbitae*^1,61–63^, *C. capitata*^64,65^, *Rhagoletis mendax* Curran^42,66^, and *Anastrepha* spp.^64,67,68^. While MRR studies allow assessment of dispersal distances for a population as limited by sampling, few studies^1,7,69^ have addressed individual pest tephritid movements in natural environments^25,30^. The prevailing assumption in the literature has been that individual step-distances are short^25,30^ (tens of meters not km) and this is supported by the current work.

Step-distances (meters per flight) for male Qflies in Experiment 1 ranged from 0.1 to 31.5 m with the most common step-distances in the papaya field being from 1 to 2 m with a mean step-distance of 3.0 ± 0.3 m (Fig. 5). This distance is shorter than that recorded with *Z. cucurbitae* in papaya in Hawaii using a similar tracking protocol*,* which had a step-distance of 6.0 ± 0.5 m^1^. This is likely due to a combinations of factors including differences in papaya tree density and canopy architecture (both were more dense in the current study), experimental protocol (steps within a tree were not recorded by Miller et al.^1^), and interspecies differences. For *B. minax,* the mean step-distances ranged from 2.3 ± 0.4 m to 6 ± 5 m depending on the experiment^7^. While in Experiment 1 flies were disrupted to keep them moving, which differs from the *B. minax* experimental protocol, mean step-distances were largely similar.

Step-distances recorded in Experiment 2 likely represent many fly movements and are therefore not directly comparable to steps recorded in Experiment 1 or in other previous HR tracking studies. However, it is interesting to note that the mean total distance moved in Experiment 2 (16 ± 5 m) is roughly half the mean path length (10–12 steps, ~ 1 h observations) in Experiment 1 (31 ± 4 m). This suggests that prodding Qflies to induce flight leads to greater dispersion than would be observed from undisturbed flies.

The relationship between step frequency vs step-distance (categorized into 1 m intervals) observed in Experiment 1 was well described by a power function (Fig. 5). This is similar to what was found with *Z. cucurbitae*^1^*,* showing that flies generally make short flights within and between nearby trees with less frequent longer flights.

Qflies tracked in Experiment 1 showed both individual-level flight directional biases (Fig. 1 and S1) and collective directional biases in turning angles (Fig. 2) but not in combined absolute flight directions (Fig. 1). At the individual level, 7 of the 20 flies observed (35%) showed directionally biased flights. Additionally, mean flight directions varied between flies (Watson-Williams test) in Experiment 1 showing strong inter-individual differences in directional orientation. These individual-level flight directional biases may be an example of biased behaviors in insects, a phenomenon increasingly described in the literature, including in the human body louse^70^, a staphylinid^71^, bumblebees^72^, 7-spot ladybird beetles^73,74^, drosophila^75^, and honeybees^76^.

The turning angle biases in individuals observed in Experiment 1 are an example of ‘persistence’ or ‘forward persistence’^77^, the tendency observed in many animals towards forward movement^78–80^. Such correlations between successive step orientations are often modeled using a CRW^77^, biased random walk (BRW, consistent bias in a preferred direction or towards a target), or biased and correlated random walk (BCRW)^80^. CRW have been observed in a number of insects^81–87^ including diptera^51^.

However, when all flight (cardinal) directions are combined and analyzed for Experiment 1, flight directions were found to be random suggesting that flies, as a group, are not orienting towards one strong directional cue (e.g. visual, light, wind cues), This is in contrast to collective biased directional movements observed with *B. minax*^7^ and *Z. cucurbitae*^1^. *Bactrocera minax* movement bias was attributed to flies moving out of an orchard and into an adjoining forest while wind was a factor in at least some cases for *Z. cucurbitae*^1^.

Insect movements in general,^88–90^ and dipterans more specifically^49^, have also been observed to follow simple random walks (i.e. with random turning angles), under certain conditions: Qfly have been reported to disperse in random directions^47^ while there has also been the suggestion that they are likely to exhibit some degree of correlated directional flight on the level of individual fly movements^48^. Evidence for dipteran RW have been observed particularly in conditions of uniform resources or environment^49,50,66^. For other tephritids, apple maggot fly movement within a tree has been shown to most closely follow a random walk with a position-dependent bias in the vertical component of the fly movements^50^. Blueberry maggot fly movement was shown to be nondirectional or random within fruit-bearing fields, having a constrained RW exhibiting directionality into the field^66^. The constraints on the RW in this case regard fly foraging outside blueberry fields, into surrounding areas, possibly by attractive visual and olfactory cues.

Combined turning angles for all step lengths in Experiment 1 (Fig. 2A) and steps greater than or equal to 0.8 m (Fig. 2B) were shown to be non-random with a pronounced bias towards forward movement. In contrast, combined turning angles for steps under 0.8 m (Fig. 2C) were random, showing no directional movement bias. This result, random directionality in shorter steps, was not observed previously while tracking *Z. cucurbitae*^1^ perhaps because of a small change in the tracking protocol. When tracking *Z. cucurbitae*, movement steps within a given tree were not recorded, thereby truncating the number of 0–1 m steps observed and effectively eliminating observations of all within-tree movements. The 0.8 m distance initially chosen for this analysis is somewhat arbitrary and was used to illustrate the break in movement directionally observed between shorter and longer steps. This initial analysis then led to the HMM and fractal analyses which allowed a more rigorous exploration of movement parameter dependence of behavioral state, spatial scale, and movement type (inter- vs intra-tree).

Experiment 2 allowed the calculation of a mean speed (1.7 ± 0.5 m/h) which is generally in line with the tens of meters/day movement generally observed in previous studies. Future tracking studies in which tagged flies are allowed to move more naturally (without artificial disturbance) are still needed.

Observed Qfly movements generally showed greater mean squared displacement than predicted by both a RW or a CRW model (Fig. 6). Mean squared displacement values showed that Qfly movements conform to the expectations of a RW or CRW model up to 4 steps. After 5 consecutive steps the mean squared displacement is greater than what would be predicted by either model. A similar positive deviation was observed by Kareiva and Shigesada with cabbage butterflies nectar-feeding in a goldenrod field^82^. This may reflect more random initial (orienting) movements that transition to directed movement based on environmental stimuli. This positive deviation also suggests that movement parameters derived from the entire data set do not adequately describe the complexity of Qfly movement at all spatial scales or for all behavioral states. That is, mean turning angles and step-distances calculated from combined data may underestimate the distances moved by Qfly due to movements that are outliers from the parameter means. Given the observation that shorter Qfly steps appeared to be correlated with more random turning angles (Fig. 2), we suspected that there might be differences between intra-tree (within tree) and inter-tree (between tree) movement behaviors and this was further investigated using HMM and fractal dimensional analysis.

While HMM have been widely used to analyze animal movements^91^, use of these models in relation to insect movement is limited but does include examples from beetle^92^ and termite^93^ studies. The two-state HMM derived in this study (Fig. 3) better fit the observed Qfly movement data from Experiment 1 than did a CRW or RW model (Fig. 7, Table S1). State 1 showed both shorter step-distances and more random turning angle when compared to state 2 (Fig. 3A,B). State 1 steps likely represent intra-tree movement while state 2 steps were generally inter-tree.

Similarly, an analysis of fractal dimension vs. spatial scale using a discontinuous two-phase linear model showed a change point in the fractal dimension at a scale of 2.48 m (Fig. 4). The fractal dimension (*D*) used in this analysis is a standardized index of movement pattern complexity permitting comparisons of patterns occurring at different scales^94^. The observation of lower fractal D (straighter movement paths) at larger spatial scales (> 2.5 m) again suggests that Qfly males move in qualitatively different manners at spatial scales below and above 2.5 m, responding differently to the surrounding microenvironment in these two regions of spatial scale. Use of fractal analysis to characterize insect movement is limited in the literature, but does include examples from grasshoppers^95^, beetles^86^, butterflies^87^, and ants^96^.

There are a limited number of previous studies using HR to track dipteran species^1,4,5,7^ likely due in part to size and not being adapted to carrying loads. It has been suggested that tag mass be kept at less than 5% of the insect body mass, though the empirical basis for this guidance is weak^4^. The other dipteran species that has been studied in the field with any depth are both tephritid fruit flies, the Chinese citrus fruit fly, *B. minax*^7^ and the melon fly^1^. Both species are larger than Qflies (approximate male weights of 44 mg and 15 mg respectively) with reported tag to fly mass ratios roughly 8% for *B. minax* and 5% for *Z. cucurbitae.* Tags on male Qflies in the current study were roughly 7% of body mass.

The movement data obtained in this study has the potential to be valuable both for Qfly control in areas where this pest fly is established and to aid in incursion management; the latter is a situation where maximal displacement is important, though difficult to measure because long distance movements are rare^97^. These data enable use of more realistic and accurate models of Qfly movement, including agent-based approaches. These models can now include rare events and use individual-movements, compared with the more commonly used diffusion models which produce distributional outcomes of the insect movement process but don’t model the movement itself^98,99^.

A limitation of this study is that tracking took place in northern Queensland during the winter months (dry season) in the southern hemisphere. Recently Tasnin et al.^100^ and Clarke et al.^101^ have presented evidence that Qflies, living outside their ancestral monsoonal rainforests, still show a pronounced seasonal reproductive arrest (not breeding during the dry season) and seasonal demographic changes (longer-lived during late autumn and late winter). Additionally, work by Dominiak et al.^102^ in the southeastern state of New South Wales has shown that wild Qflies have lower body masses during the tracking period of the current study. This seasonal phenology may cause insects in reproductive diapause to have greater stress resistance, cold tolerance, and perhaps altered movement. As searching for mates is a primary driver for male movement, male Qflies may be expected to decrease movement activity when females are unreceptive to mating. In light of the work conducted by Tasnin et al.^100^ and Clarke et al.^101^, it would be pertinent to conduct further Qfly movement tracking during summer and autumn to assess if movement parameters change when mating levels are expected to be highest.

This study involved tracking wild male fruit flies. Wild male fruit flies are easy to catch in male lure traps, however, catching wild females is very difficult, especially during the dry season in north Queensland when populations are very low. Future research will endeavor to track immature females that have been reared from infested fruit and will further expand on the models that have been developed in this paper.

This study demonstrates the feasibility of tracking individual Qfly, a highly mobile, medium-sized flying insect using small, light-weight HR tags with flexible antennas. Observed Qfly step-distances and turning angles from Experiment 1 were similar to those reported for *Z. cucurbitae*^1^ which were recently tracked using similar techniques. Using movement parameters from Experiment 1, RW, CRW, and HMM models were used to simulate Qfly movements. Experiment 2 identified individual Qfly landing locations twice a day demonstrating that flies could be tracked over multiple days. Movement parameters determined in this study provide data which may help enhance current surveillance, control, and eradication methods, such as optimizing trap placements and pesticide applications, determining release sites for parasitoids, and setting quarantine boundaries after incursions.

## Materials and methods

### Insects

Wild male *B. tryoni* were collected on the grounds of the Department of Agriculture and Fisheries facility in Mareeba, QLD, Australia (−17.007706, 145.430037), using Lynfield traps baited with cuelure (4-(3-oxobutyl)phenyl acetate). Traps were hung at a height of approximately 1.8 m at least 5 m apart (vegetation permitting). Flies were collected daily at approximately 10:00 am. Flies were trapped, tagged, and tracked within the same day. Flies that were collected but not immediately tagged were held in BugDorm-4F3030 insect rearing cages (32.5 cm × 32.5 cm × 32.5 cm) and supplied with water and sugar cubes in a constant temperature laboratory (26 ± 1 °C, ~ 70% RH, natural light ~ 11L:13D). Flies held for more than 24 h were not tagged and released. Additionally, flies that failed to exhibit flight behavior in cages after initial capture were not tagged.

After the desired number of flies were set aside for tracking on a particular day, the remaining flies were immediately frozen. Tracked flies that were recaptured after ~ 10 steps, as well as unreleased tagged flies, were also frozen. Mean fly mass was determined by weighing groups of frozen flies.

### Harmonic radar tag fabrication and attachment

Dipole harmonic radar tags were fabricated from a Schottky diode (RECCO AB, Lidingö, Sweden) and straight annealed 0.0254 mm diameter superelastic nitinol wire purchased from Fort Wayne Metals (Fort Wayne, IN, USA) as outlined by Miller et al.^1^ Briefly, two 4 cm lengths of wire were attached to the diode with UV activated adhesive (Bondic, Niagara Falls, NY, USA). Electrical connections between the wires and the diode contacts were secured using conductive silver paint (GC Electronics, Rockford, IL, USA). Individual tags weighed approximately 0.8 mg. The signal strength of each tag was tested after assembly using a harmonic radar transceiver unit (R9) purchased from RECCO. Tags that returned the strongest signals were subsequently attached to flies for use in tracking.

To prepare for tag attachment, flies were immobilized in a freezer for 1 min or until cessation of movement. Individual flies were then held by the legs and a tag, dipped in the UV activated adhesive, was positioned in a longitudinal orientation on the dorsal surface of the thorax before being cured with light from a UV LED. Care was taken not to glue the wings or the head during tag attachment.

### General tracking protocol

Locating flies in the field with HR was accomplished either by searching an area to which a fly was visually noted to have flown to (sometimes possible in Experiment 1) or by searching throughout the study field area in a regular pattern. In Experiment 1, if a potential landing site was detected visually, the surrounding trees and ground were searched for a signal for the first 2 min and then subsequent rows were methodically swept using the RECCO transceiver. During searching, the RECCO unit was rotated and moved from side to side to maximize signal detection by aligning the transceiver with the tag attached to the fly. Under optimal conditions, alignment of the RECCO unit with the tag, without vegetation interference, yielded a maximum detection range of approximately 20 m with a strong signal generally detected at approximately 10 m. However, in the papaya field under field tracking conditions, detection distances were closer to 3 m due to suboptimal alignment of tags with the transceiver and interference from vegetation.

When a signal was found, the time was recorded, and the tree was searched. Once a visual of the fly had been obtained, a second time was recorded, and the location was marked using flagging tape. If a strong signal was found and the fly took flight before a visual was made the suspected location was still flagged as a step based on the strong signal. The length of the steps was recorded at ground level and the direction was marked for each step using a compass.

### Study site

A subsection of larger papaya field in Paddy’s Green, QLD, Australia (several hundred meters surrounding the release point: −16.975923, 145.310677) was used for both experiments 1 and 2. The release point was a PVC podium with a roughly 30 cm^2^ surface area mounted on a roughly 40 cm length of PVC placed between several papaya trees. The study area size was searchable in 20 min. Papaya trees in the study area were planted in raised double rows with approximately 3 m between trees (Fig. 2S). Each double row of trees was separated by a roughly 2 m wide dirt/mowed-grass access track. Trees ranging in height from 2.5 to 3.5 m with the foliage of one tree nearly touching that of the neighboring tree within a row. Trees were bearing fruit during the experimental period. Ground cover plants were short (generally less than 30 cm) and sparce throughout the field. The study field area was bordered on west, south, and east by a dirt/gravel access road with sporadic windbreak trees approximately 100 m from the release point. Areas outside of the papaya field were not searched during either experiment.

Experiments were conducted between 9:30 am and 4:30 pm. Weather conditions during this time were generally sunny with a mean temperature of 22.8 °C and a mean wind speed of 8.2 m/s generally blowing from the southeast (data from weather station at −16.99, 145.37). Several attempts were made to record weather data at the release point and at various locations in the study field. Unfortunately, due to instrument failures and experimental difficulties, the data recorded was too unreliable to use in further analyses.

### Experiment 1—Continuous Observation (16 June 2022—8 July 2022)

Experiment 1 investigated the continuous movement of tagged Qflies in the papaya study field over 10–12 steps (flights). Tagged flies were released one at a time from the PVC podium. If a fly did not take off within 5 min of being released, a piece of grass was used to encourage flight. If no flight occurred with prodding, the fly was designated a nonflier, collected, and placed in a separate cage.

Flies were tracked one at a time after being released into the field. After release, tagged flies were tracked through the field with landing locations (specific tree) recorded after each flight. At most landing locations the fly was visually located, however, in some instances the fly took flight before a visual confirmation was possible and the presence of the fly was identified by signal detection only. Flies were allowed to rest for 5 min following each flight. If a fly had not flown again after 5 min, the surrounding foliage was disturbed to induce flight. All flights were recorded, even flights between leaves within a tree. Flies with at least five recorded flights were used in the analysis. Up to 12 steps were recorded for each tagged fly. When possible, flies were recaptured and removed from the field after 10–12 recorded flights. Step-distances (flight distances), flight directionality (angle from take-off to landing), and turning angles (angle between successive flight directions) were calculated from recorded fly positions. Tracking a single fly until 10–12 steps generally required about an hour. The number of flies tracked per day ranged from 2 to 5.

### Experiment 2—Periodic Observation (11–12 July 2022 and 18 July 2022)

Experiment 2 was designed to investigate the natural (undisturbed) movement of tagged Qflies over the course of several days. Flies were collected, tagged and released at the same time/place at the beginning of the tracking period, and tracked twice a day. In order to release flies at the beginning of the day, wild male flies were captured in the morning, tagged, and then released into the study field site midmorning (10:00–12:00).

For this experiment, tag diodes were painted different colors using nail polish to allow visual identification of specific flies in the field. A roughly square 120 m by 120 m area was flagged in the same study site area of the papaya field where previous tracking was conducted. The first trial began on 11 July 2022 and consisted of seven tagged flies being released from the release point at the center of the plot and subsequently tracked. During this first trial, the trees surrounding the release point were searched first before spreading out and systematically searching every row of the study area.

For the second trial (begun 18 July 2022) ten flies were released and to reduce bias, searching was conducted by systematically searching from the west of the study area to the east. When a signal was detected, the fly was sighted and removed from the tree to check the color of the tag, thus identifying the fly. The location was flagged with the tag color, signal time and sighted time, before the fly was placed back on the exact location. The sweeping process was repeated twice a day (morning and afternoon) until no signals were detected when the entire plot was scanned. After each pass through the area, distance and direction was measured from the central release point to flagged landing locations.

### Statistical analysis

For Experiments 1 and 2, the Watson-Williams test for homogeneity of means was used to determine if the flight directions varied between flies. Subsequently the Rayleigh test and the Hermans–Rasson test^103^ were used to determine if flight directions were random for each set of flights for an individual fly and for all flies taken together. Comparisons of turning angles grouped into 45° quadrants were carried out using chi-squared analyses in R^104^. All circular statistical analyses were performed using R packages CircStats^105^, circular^106^, and CircMLE^107^.

Equations for step frequencies vs. step-distances were calculated using Microsoft Excel (Version 2108, Microsoft Corp. Redmond WA USA). The mean movement rate was calculated by averaging the distance moved (m) between two observation points divided by the time (h) between the two observations.

Mean squared displacements (MSD) for Qfly movements in Experiment 1 along with MSD predicted by a CRW model were calculated using Fractal 5 (version 5.26.0)^108^. Deviations in MSD between observed Qfly movements and those predicted by the CRW model were also tested using Fractal 5^109^. Predicted MSD for a RW were calculated using the following equation,

$${\text{MSD }} = \, nL$$

where *n* = number of consecutive, *L* = mean squared move length (m^2^)^51,82^.

Analyses of fractal dimension at different spatial scales were performed using Fractal 5 following Nams and Bourgeois^109^. Fractal dimensions were estimated using the following parameters: window range was set at 0.35, spatial scales ranged from 1 to 50 m, and the number of divisions was set to 100. Initial analyses of fractal dimension vs. spatial scales showed marked variations attributable to the varying path lengths included in each estimation of the fractal dimension. To control for this variation, two short paths were dropped from the analysis and only spatial scales that included all remaining eighteen paths, ~ 1.4 to 10 m, were included. Threshold regression analyses were performed on log transformed data using the R package chngpt^110^. Both continuous and discontinuous two-phase models were fitted using the segmented and stegmented commands, respectively.

Qfly movement models and simulations were performed using the R packages adehabitatLT^111^, moveHMM^112^, and aniMotum^113^. Initial parameters for the two-state hidden Markov model (HMM) were varied to avoid the optimizer estimates converging to a local maximum^114^. Example simulations with RW, CRW, and HMM were constructed for 20 flies, 10 steps each (to mimic field data) and 100 flies, 100 steps (to compare movement predictions for each model. Comparisons between mean distances moved in simulations were conducted by ANOVA by means comparisons with Tukey’s HSD (*α* = 0.05).

### Supplementary Information

Supplementary Information 1.

Supplementary Information 2.

Supplementary Information 3.

Supplementary Information 4.

Supplementary Information 5.

## Data Availability

The data that support the findings of this study are available from the corresponding author upon reasonable request. There are no restrictions on data availability.
