# Supplementary figures and images for: Tracking and modeling the movement of Queensland fruit flies, Bactrocera tryoni, using harmonic radar in papaya fields
Source: Sci Rep. 2024 Jul 30;14:17521. doi: 10.1038/s41598-024-67372-4 (PMC11289093; doi:10.1038/s41598-024-67372-4)

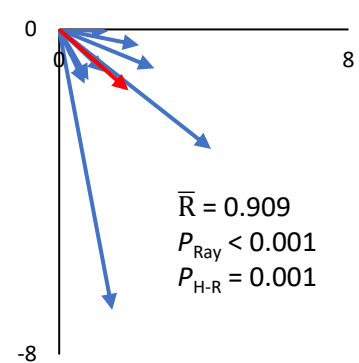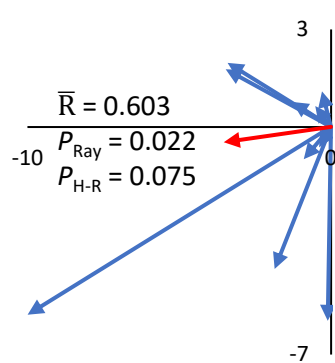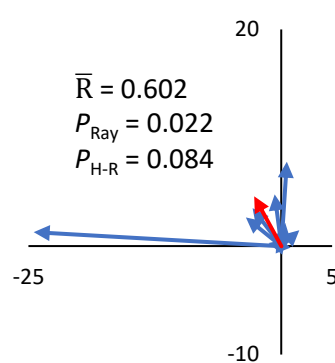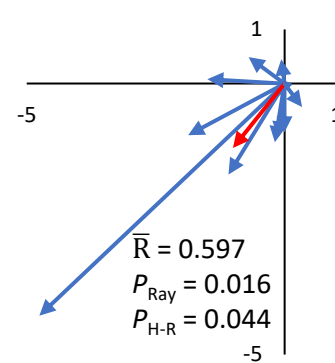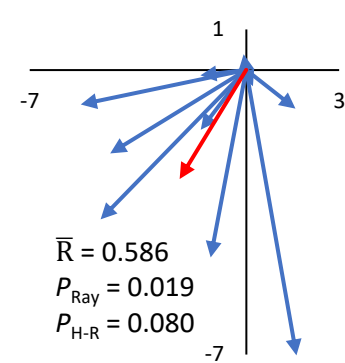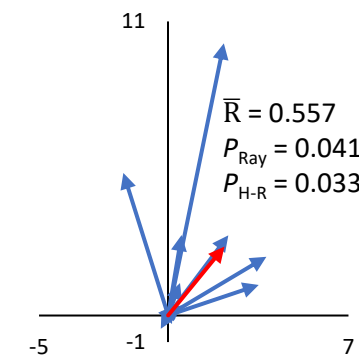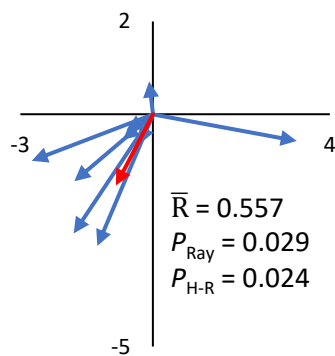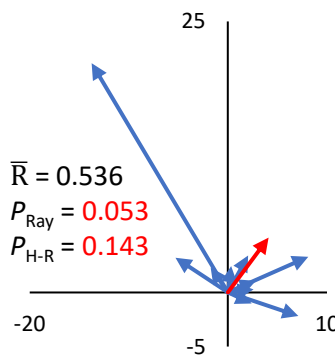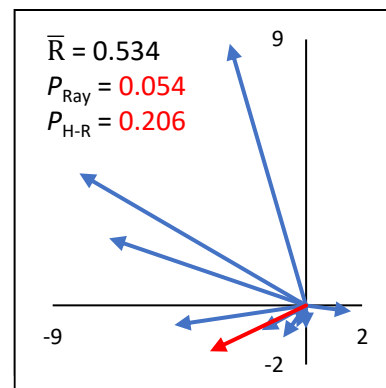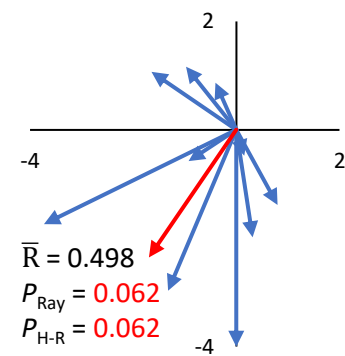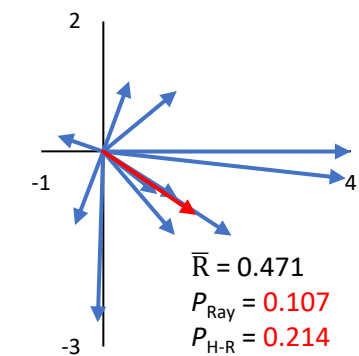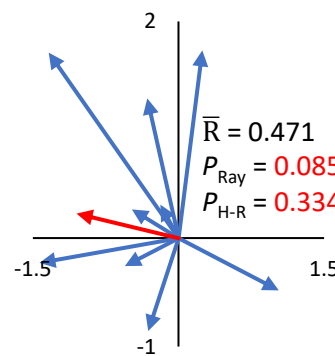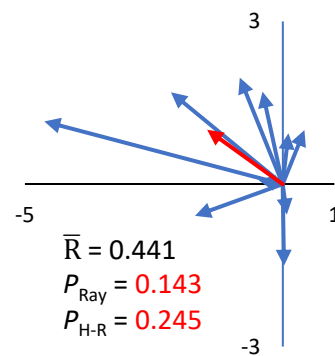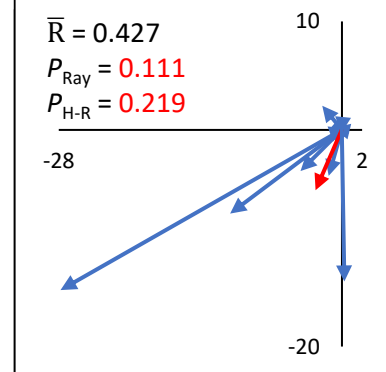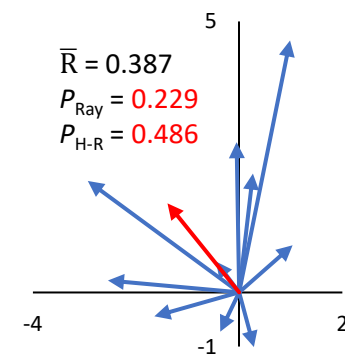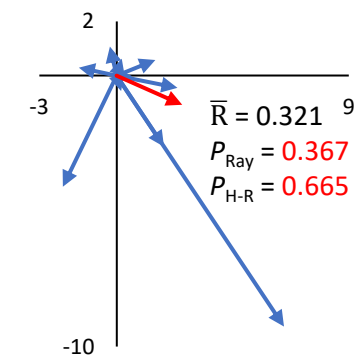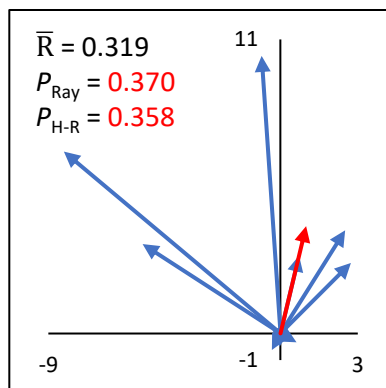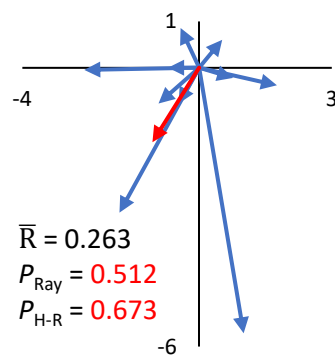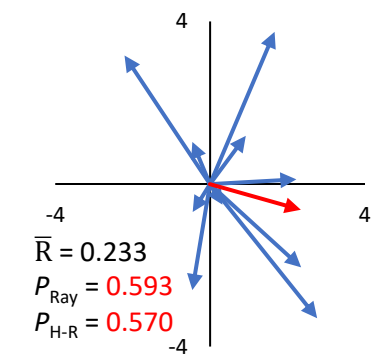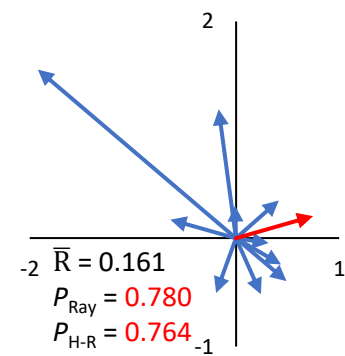

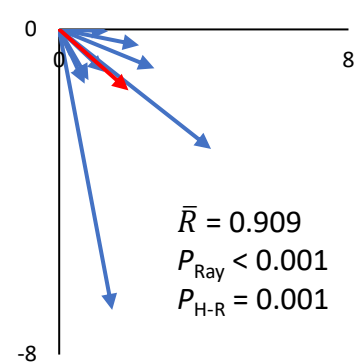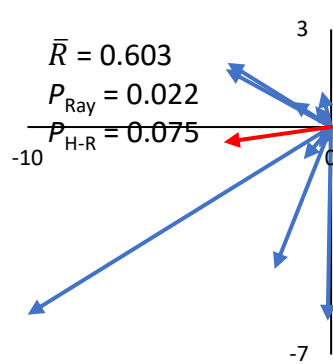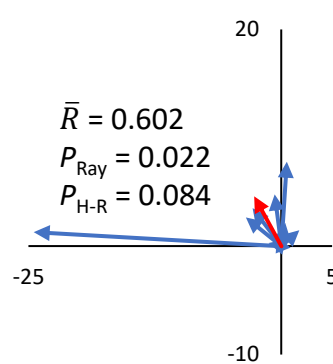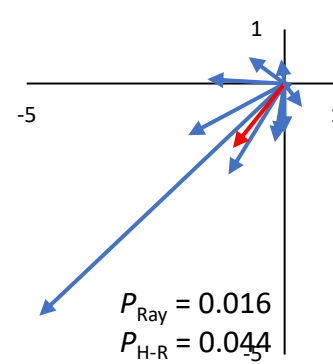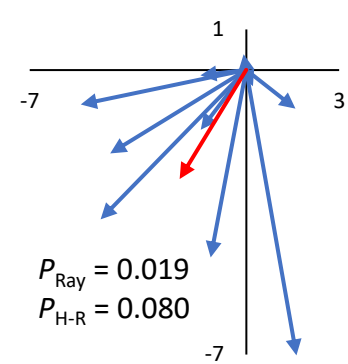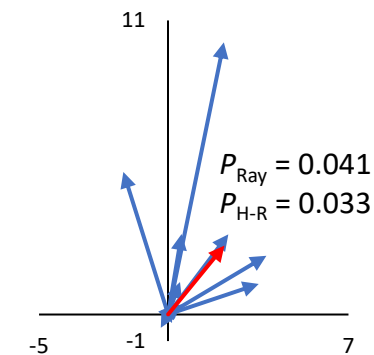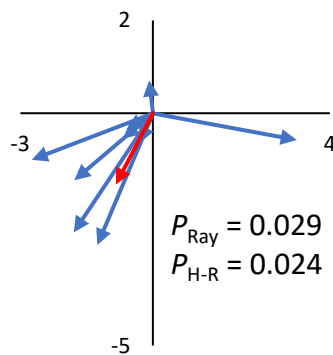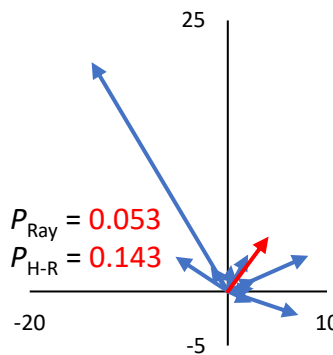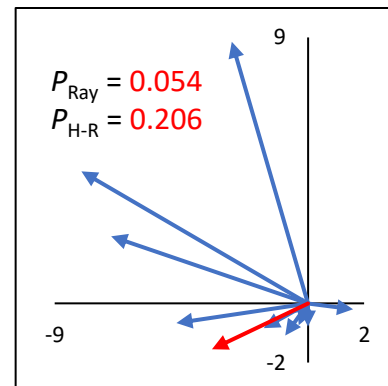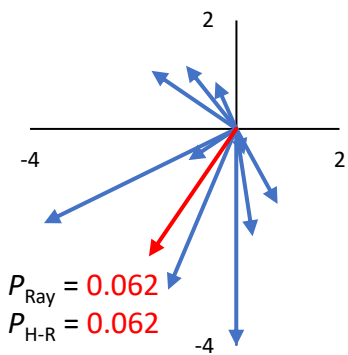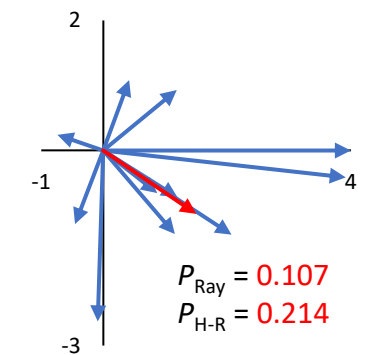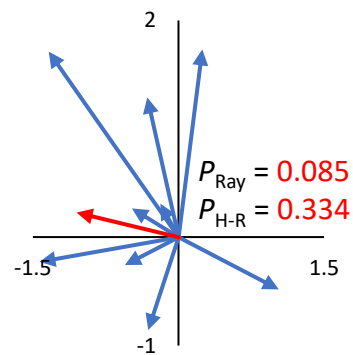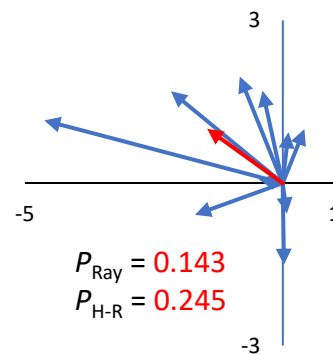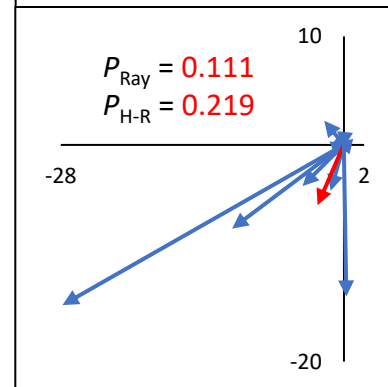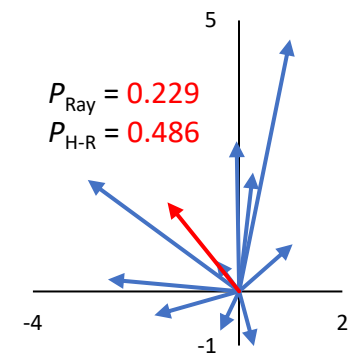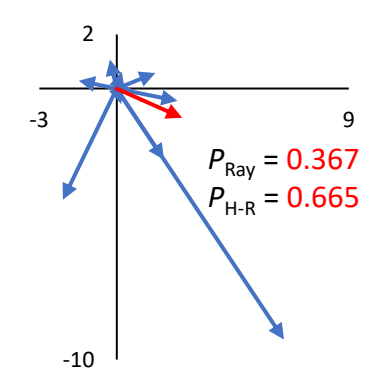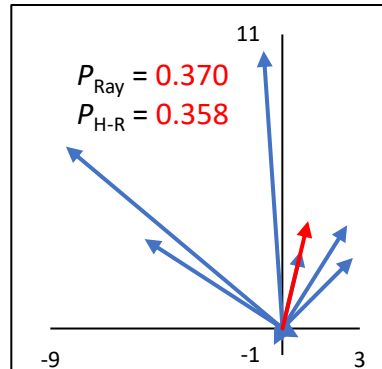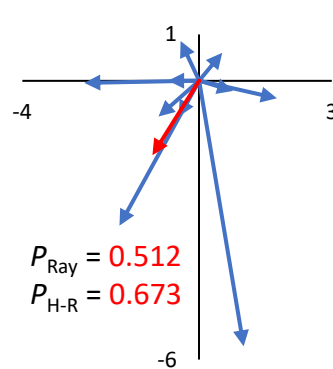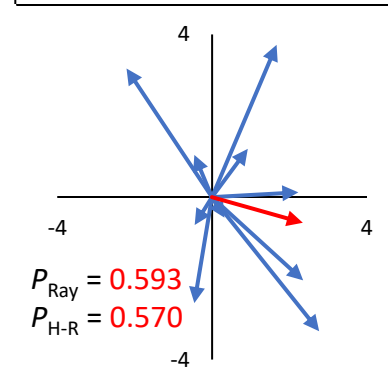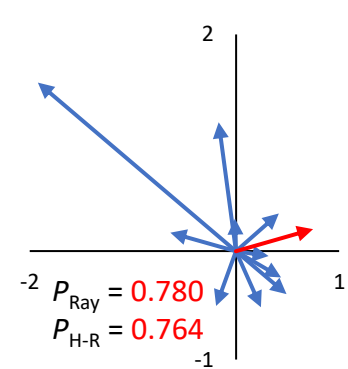

Supplement: Supplementary file 1 — Supplementary Information 1. [file 41598_2024_67372_MOESM1_ESM.pdf]

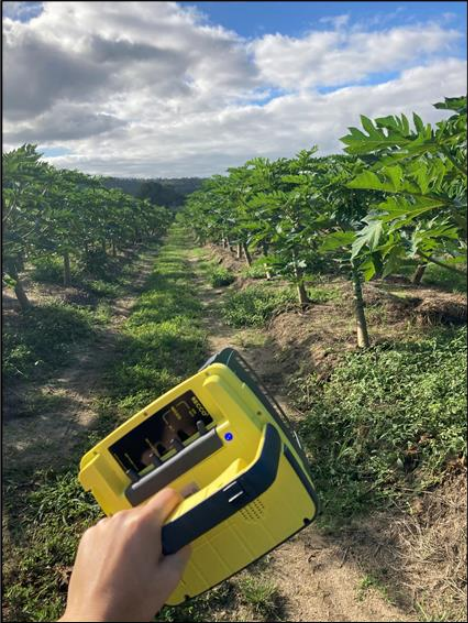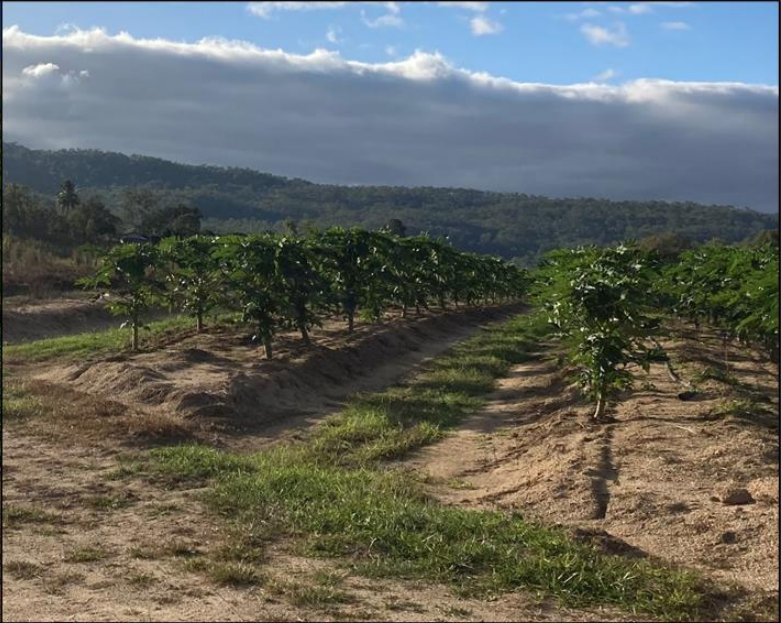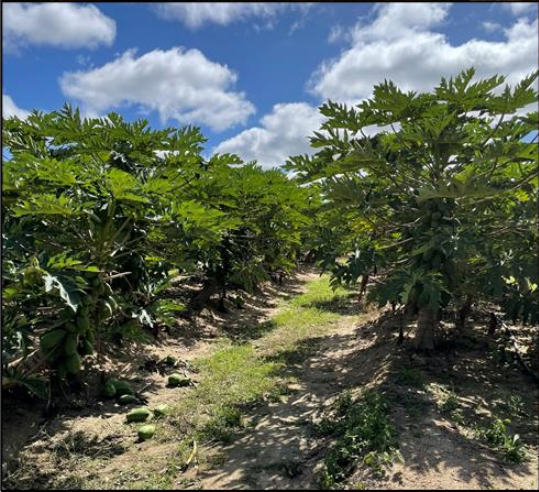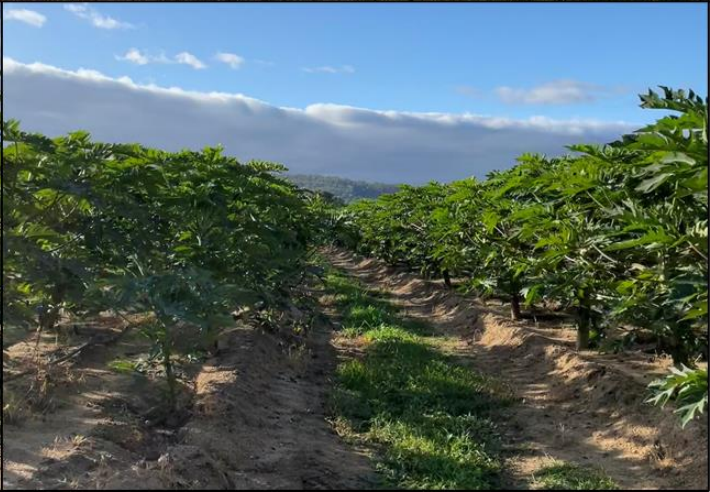

Supplement: Supplementary file 2 — Supplementary Information 2. [file 41598_2024_67372_MOESM2_ESM.pdf]
