# Supplementary material for: Tracking and modeling the movement of Queensland fruit flies, Bactrocera tryoni, using harmonic radar in papaya fields
Source: Sci Rep. 2024 Jul 30;14:17521. doi: 10.1038/s41598-024-67372-4 (PMC11289093; doi:10.1038/s41598-024-67372-4)
Supplement: Supplementary file 3 — Supplementary Information 3. [file 41598_2024_67372_MOESM3_ESM.docx]

**Figure S1.** HR tagged *Bactrocera tryoni* flight directions and lengths for Experiment 1 (induced movement). Each replicate consisted of a series of 10-12 flights with a single tagged fly. Blue arrows represent individual flights, while red arrows show the mean flight direction and length. Black borders highlight flies that had directionally correlated long movements and shorter uncorrelated movements resulting in uncorrelated movement overall.

**Figure S2.** Experimental site showing typical foliar density and access track width.
