# Supplementary material for: Tracking and modeling the movement of Queensland fruit flies, Bactrocera tryoni, using harmonic radar in papaya fields
Source: Sci Rep. 2024 Jul 30;14:17521. doi: 10.1038/s41598-024-67372-4 (PMC11289093; doi:10.1038/s41598-024-67372-4)
Supplement: Supplementary file 4 — Supplementary Information 4. [file 41598_2024_67372_MOESM4_ESM.docx]

**Table S1.** Akaike information criterion (AIC) values evaluating the model fit for the movement data of each individual *Bactrocera tyroni* tracked in Experiment 1. The AICs for HMM and CRW models with all Qfly movements are 1486.18 and 1518.58, respectively.

|  | **AIC** | |
| --- | --- | --- |
| **Fly ID** | **RW** | **CRW** |
| F1 | **-149.5** | **-95.6** |
| F2 | **-127.8** | **-44.6** |
| F3 | **-94.4** | **Inf** |
| F4 | **-141** | **-90** |
| F5 | **-132.2** | **-66.5** |
| F6 | **-178.1** | **-125.1** |
| F7 | **-157.7** | **-99.2** |
| F8 | **-155.7** | **-94.7** |
| F9 | **-169.2** | **-104.7** |
| F10 | **-175.9** | **-171.4** |
| F11 | **-171.4** | **-120.4** |
| F12 | **-114.3** | **-79.3** |
| F13 | **-195.9** | **-178.5** |
| F14 | **-136** | **-70.1** |
| F15 | **-101.7** | **-50.9** |
| F16 | **-166.7** | **-106.3** |
| F17 | **-152** | **-102.2** |
| F18 | **-128.5** | **-41.5** |
| F19 | **-172.8** | **-130.6** |
| F20 | **-190.4** | **-151.3** |
