# Supplementary material for: Tracking and modeling the movement of Queensland fruit flies, Bactrocera tryoni, using harmonic radar in papaya fields
Source: Sci Rep. 2024 Jul 30;14:17521. doi: 10.1038/s41598-024-67372-4 (PMC11289093; doi:10.1038/s41598-024-67372-4)
Supplement: Supplementary file 5 — Supplementary Information 5. [file 41598_2024_67372_MOESM5_ESM.docx]

**Table S2.**  Distances (mean ± SE) moved by Qflies from the origin in simulations of 100 flies taking 100 steps each. 95% and 50% distances indicate the mean radius of a circle that encompasses 95% and 50% of movements, respectively. Means with different letters are significantly different (ANOVA followed by mean separation with Tukey’s HSD, α = 0.05).

|  | RW |  | CRW |  | HMM |
| --- | --- | --- | --- | --- | --- |
| max dist (m) | 109 ± 3 c |  | 188 ± 2 a |  | 130 ± 2 b |
| 95% dist (m) | 54.6 ± 0.5 c |  | 98 ± 1 a |  | 67.0 ± 0.5 b |
| 50% dist (m) | 23.0 ± 0.3 c |  | 39.2 ± 0.2 a |  | 26.3 ± 0.2 b |
